# Reducing Caloric Intake Prevents Ischemic Injury and Myocardial Dysfunction and Affects Anesthetic Cardioprotection in Type 2 Diabetic Rats

**DOI:** 10.1155/2017/4126820

**Published:** 2017-02-28

**Authors:** Charissa E. van den Brom, Christa Boer, Rob F. P. van den Akker, Stephan A. Loer, R. Arthur Bouwman

**Affiliations:** ^1^Experimental Laboratory for Vital Signs (ELVIS), Department of Anesthesiology, VU University Medical Center, Amsterdam, Netherlands; ^2^Department of Physiology, VU University Medical Center, Amsterdam, Netherlands

## Abstract

*Background*. Type 2 diabetes mellitus (T2DM) increases the risk of myocardial ischemia, followed by increased perioperative risk of cardiovascular morbidity. We investigated whether reducing caloric intake reduces ischemic injury and myocardial dysfunction and affects the protective effects of the volatile anesthetic sevoflurane in diet-induced T2DM rats.* Methods*. Rats received a western (WD) or control diet (CD). Caloric intake was reduced by reversing WD-fed rats to CD. Myocardial function was determined with echocardiography. After 8 weeks of diet feeding, myocardial infarction was induced and the effect of sevoflurane was studied on myocardial function and ischemia/reperfusion injury.* Results*. WD-feeding resulted in a mild T2DM phenotype and myocardial dysfunction. Sevoflurane further impaired systolic function in WD-fed rats. Unexpectedly, WD-feeding reduced infarct size compared to CD-feeding. Sevoflurane reduced infarct size in CD-fed rats; however it enlarged infarct size in WD-fed rats. Caloric reduction restored myocardial dysfunction and the protective effect of sevoflurane against ischemia compared to WD-fed rats, whereas the protective effects of WD-feeding persisted.* Conclusion*. Caloric reduction restored the T2DM phenotype and myocardial function, while the cardioprotective properties of WD-feeding or sevoflurane persisted. Our data suggest that reducing caloric intake in T2DM might be a possible intervention to reduce perioperative risk of cardiovascular morbidity.

## 1. Introduction

The prevalence of patients with type 2 diabetes (T2DM) is still increasing worldwide and also anesthetists increasingly face these patients. Patients with T2DM are more likely to develop coronary artery disease and myocardial ischemia [1], which further increases the perioperative risk of myocardial ischemia [2].

Excessive caloric intake and a sedentary lifestyle contribute to the development of T2DM. Reducing caloric intake reverses diet-induced obesity in rodents [3, 4]; therefore, this may be an attractive approach to reduce perioperative risk. Unfortunately, there is only limited literature available that describes the effects of caloric restriction on myocardial function and ischemic injury. In patients, a low caloric diet decreased myocardial fatty acid uptake [5] and improved diastolic function [6], suggesting that reduction of caloric intake might also have positive effects on ischemic injury.

Another aspect that influences perioperative myocardial function and ischemic injury is anesthesia. The volatile anesthetic, sevoflurane, is known to depress myocardial contractility [7], impair diastolic function [7, 8], and decrease systemic vascular resistance [8]. These cardiovascular effects may not be harmful in healthy subjects, but patients with T2DM might be at increased risk due to loss of compensatory mechanisms. In diabetic rats, we already showed that myocardial function is impaired and that sevoflurane even further impaired systolic function [9].

Besides cardiodepressive effects, sevoflurane also exerts cardioprotective effects as shown by reduced myocardial ischemic injury in rats [10, 11]. However, in obese [12], hyperglycemic [13], and insulin resistant [14] rats, sevoflurane-induced cardioprotection was blocked, suggesting that obesity and/or T2DM influences these cardioprotective properties negatively. Taken together, it seems that obesity and T2DM have detrimental effects on myocardial function and ischemic injury; however, the question remains if this could be improved by reducing caloric intake. Therefore we investigated whether reducing caloric intake reduces myocardial dysfunction and ischemic injury and potentiates the cardioprotective effects of sevoflurane anesthesia in diet-induced T2DM rats.

## 2. Materials and Methods

### 2.1. Animals and Experimental Set-Up

This study was carried out in strict accordance with the European Convention for the Protection of Vertebrate Animals used for Experimental and Other Scientific Purposes. All experiments were approved by the Institutional Animal Care and Use Committee of the VU University (permit numbers: FYS 10-08 and ANES 12-06) and performed in compliance with the modern ARRIVE guidelines on animal research [15]. All surgeries were performed under S-ketamine and diazepam anesthesia and analgesics, and all efforts were made to minimize suffering.

**Figure 1:**
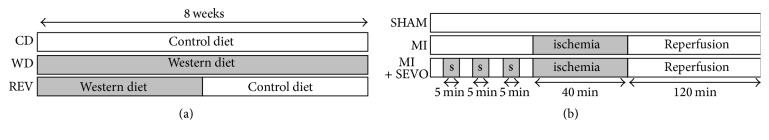
Experimental protocol. Rats were fed a control diet (CD) or western diet with sucrose water (WD) for 8 weeks. A third group of WD-fed rats reversed after 4 weeks to CD for 4 consecutive weeks (REV) (a). After 8 weeks of diet exposure, animals underwent left coronary artery occlusion for 40 min followed by 120 min of reperfusion without (MI) or with 3 × 5 min sevoflurane (s) exposure (MI + SEVO) (b).

The study was divided into two parts. The first part consisted of 21 male Wistar rats (baseline body weight: 264 ± 5 g; Charles River Laboratories, France), which were exposed to a western diet in combination with sucrose water (20%) (WD) or control diet (CD) for a period of 8 weeks. Four weeks after the start of diet exposure, 50% of the WD-fed rats reversed to CD for 4 weeks (REV) (Figure 1(a)). After 4 and 8 weeks of diet exposure, the diet-induced phenotypes were characterized by an oral glucose tolerance test and echocardiography to determine glucose tolerance and myocardial function, respectively. Additionally, systolic function was determined with echocardiography under baseline conditions and after sevoflurane exposure.

The second part of the study consisted of 144 male Wistar rats (baseline body weight: 263 ± 1 g) and received the same diets as described in part 1. After 8 weeks of diet exposure, a myocardial infarction was induced followed by reperfusion. Additionally, the effect of sevoflurane on ischemia and reperfusion injury was studied.

All rats were housed in a temperature-controlled room (20–23°C; 40–60% humidity) under a 12/12 h light/dark cycle starting at 6.00 am. Body weight was determined on a weekly basis.

### 2.2. Diets

Control diet (Teklad 2016, Harlan, Horst, Netherlands) consisted of 20% kcal protein, 9% kcal fat, and 74% kcal carbohydrates (1804 kcal/kg starch, 200 kcal/kg sugars), whereas western diet (D12451, Research Diets, New Brunswick, NJ) consisted of 20% kcal protein, 45% kcal fat, and 35% kcal carbohydrates (291 kcal/kg starch, 691 kcal/kg sugars) with 20% sucrose water (800 kcal/kg), totally containing 3300 kcal/kg and 4857 kcal/kg for CD and WD with sucrose water, respectively.

### 2.3. Oral Glucose Tolerance Test

After four and eight weeks of diet feeding, awake rats received an oral glucose load (2 g/kg of body weight) after overnight fasting. Blood glucose was measured from tail bleeds with a Precision Xceed Blood Glucose monitoring system (MediSense, UK) before and 15, 30, 60, 90, and 120 min after glucose ingestion. At similar time points, plasma insulin (LINCO research, St. Charles, Missouri) levels were measured [16].

### 2.4. Echocardiography

Echocardiography (ALOKA ProSound SSD 4000, ALOKA, Tokyo, Japan) was performed after 4 and 8 weeks as described previously [9, 16, 17]. Briefly, rats received S-ketamine (Ketanest®, 125 mg/kg, Pfizer, Netherlands) and Diazepam (4 mg/kg, Centrafarm, Netherlands) intraperitoneally and maintenance was performed with 40 mg/kg/h S-ketamine and 1 mg/kg/h diazepam intravenously. Left ventricular (LV) dimensions during end-systole (ES) and end-diastole (ED) were determined in the Motion mode of the parasternal short-axis view at the level of the papillary muscles. LV systolic function is represented by fractional shortening (FS) and fractional area change (FAC), which were calculated by FS = ((EDD − ESD)/EDD · 100) and FAC = ((EDD^2^ − ESD^2^)/EDD^2^ · 100). Diastolic function was measured in the apical four-chamber view and shown as E deceleration time and isovolumic relaxation time (IVRT; except 8 weeks data). LV mass was calculated as described previously [17]. After baseline measurements, rats were exposed to 2% (v/v) sevoflurane in 40% O_2_/60% N_2_ for 5 minutes, whereas control rats received only 40% O_2_/60% N_2_, followed by measurements of systolic function. All parameters were averaged over at least three cardiac cycles. Analyses were performed off-line (Image-Arena 2.9.1, TomTec Imaging Systems, Germany).

### 2.5. Blood and Plasma Measurements

After 6 hours fasting, rats were sacrificed and trunk blood was collected for plasma determinations. Plasma hematocrit levels were determined using microcentrifugation. Plasma glucose (Abcam, Cambridge, MA), insulin (LINCO research, St. Charles, Missouri), free fatty acids (WAKO NEFA-HR, Wako Pure Chemical Industries, Osaka, Japan), triglyceride (Sigma, Saint Louis, Missouri), and plasma HDL and LDL/VLDL cholesterol (Abcam, Cambridge, MA) levels were measured from trunk blood [9, 16, 17].

### 2.6. Myocardial Infarction

Rats from the second part of the study were anesthetized with 125 mg/kg S-ketamine (Ketanest, Pfizer, Netherlands) and 4 mg/kg diazepam (Centrafarm, Netherlands) intraperitoneally. Trachea was intubated and lungs mechanically ventilated (positive end-expiratory pressure, 1-2 cm H_2_O; respiratory rate, ~65 breaths/min; tidal volume, ~10 mL/kg) with oxygen-enriched air (40% O_2_/60% N_2_). Anesthesia was maintained by continuous infusion of 40 mg/kg/h S-ketamine and 1 mg/kg/h diazepam intravenously [9]. The respiratory rate was adjusted to maintain pH and carbon dioxide within physiological limits. Body temperature was maintained stabile (37.1 ± 0.05°C) by using a warm water underbody heating pad. The left carotid artery was cannulated for blood sampling for blood gas analyses (ABL50, radiometer, Copenhagen, Denmark) and for measurements of arterial blood pressure. Arterial blood pressure, ECG, and heart rate were continuously recorded using PowerLab software (Chart 7.0; ADInstruments, Castle Hill, Australia).

Left thoracotomy was performed between the fourth and fifth rib. A ligature (Prolene® 6-0; Ethicon, San Lorenzo, Puerto Rico) was placed around the left anterior descending coronary artery (LAD) with a special ligation device that allowed pulling the suture in one simple movement in order to reduce the chance of preconditioning [18]. Successful coronary occlusion was verified by ECG changes. Ischemia was maintained for 40 min and followed by 120 min of reperfusion (MI) (Figure 1(b)). Rats in the control (CON) group received a similar surgical preparation procedure, but without LAD occlusion. Sevoflurane intervention was induced by three periods of 5 minutes exposure to 2% (v/v) sevoflurane, interspersed with washout periods of 5 minutes before ischemia and reperfusion (MI + SEVO).

### 2.7. Evans Blue and TTC Staining

After 120 min of reperfusion, hearts were excised and the aorta was cannulated. The LAD was reoccluded and 0.2% Evans Blue dye (Sigma, St. louis, MO) was infused to stain the nonischemic myocardium leaving the area at risk unstained. After rinsing with 0.9% NaCl, hearts were stored at −20°C. For determination of infarct size, frozen hearts were cut in slices of 1 mm, incubated in 2,3,5-triphenyl tetrazolium chloride (TTC; Sigma, St. Louis, MO) solution at 37°C for 15 min, followed by fixation in 4% formaldehyde (Klinipath, Duiven, Netherlands). Slices were scanned and the area at risk and infarct size were determined using ImageJ (1.42q, National Institute of Health). The infarct size is presented as the percentage of the area at risk.

### 2.8. Statistical Analysis

Data were analyzed using Graphpad Prism 5.0 (La Jolla, USA) and presented as mean ± SD. Statistical analyses were performed using Student's *t*-test (4 weeks of diet exposure), one-way ANOVA with Bonferroni post hoc analysis (8 weeks of diet exposure), and two-way ANOVA with Bonferroni post hoc analysis (with repeated measurements) for additional interventions. *p* < 0.05 was considered as statistically significant.

## 3. Results

### 3.1. Reduction of Caloric Intake Normalized Western Diet-Induced Mild Type 2 Diabetic Phenotype

**Figure 2:**
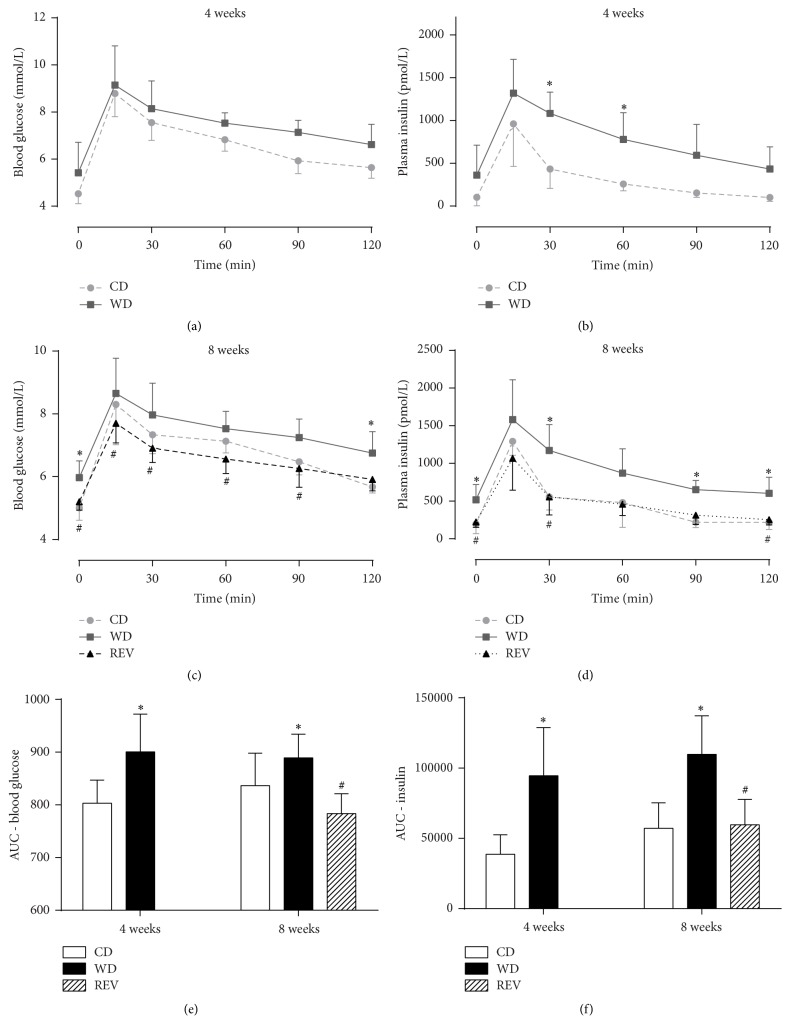
Glucose tolerance after diet feeding without sevoflurane exposure. Blood glucose (a, c), insulin (b, d) levels, and area under the curve (AUC; e, f) during an oral glucose tolerance test in rats after 4 and 8 weeks of control diet (CD), western diet (WD), or diet reversion (REV) feeding. Data are mean ± SD, *n* = 6, Student's *t*-test, one- and two-way ANOVA with repeated measures, and Bonferroni post hoc analyses, ^*∗*^*p* < 0.05 versus CD and ^#^*p* < 0.05 versus WD.

Characteristics of rats after 4 weeks and 8 weeks of diet intervention are summarized in Table 1. At 4 weeks, rats fed a WD had significantly increased body weight, plasma insulin, and triglyceride levels and reduced HDL cholesterol levels compared to rats fed a CD. After 8 weeks also plasma glucose and LDL/VLDL levels were significantly increased when compared to controls. Moreover, postload blood glucose and plasma insulin levels were increased in WD-fed rats when compared to control rats (Figure 2), suggesting that 4 and 8 weeks of WD-feeding resulted in a mild T2DM phenotype. Switching back to control diet after 4 weeks of WD-feeding resulted in a significant lower caloric intake and normalization of obesity, hyperinsulinemia, hyperglycemia, and mild dyslipidemia and improved glucose tolerance of WD-fed rats.

### 3.2. Improved Myocardial Function after Reducing Caloric Intake

**Figure 3:**
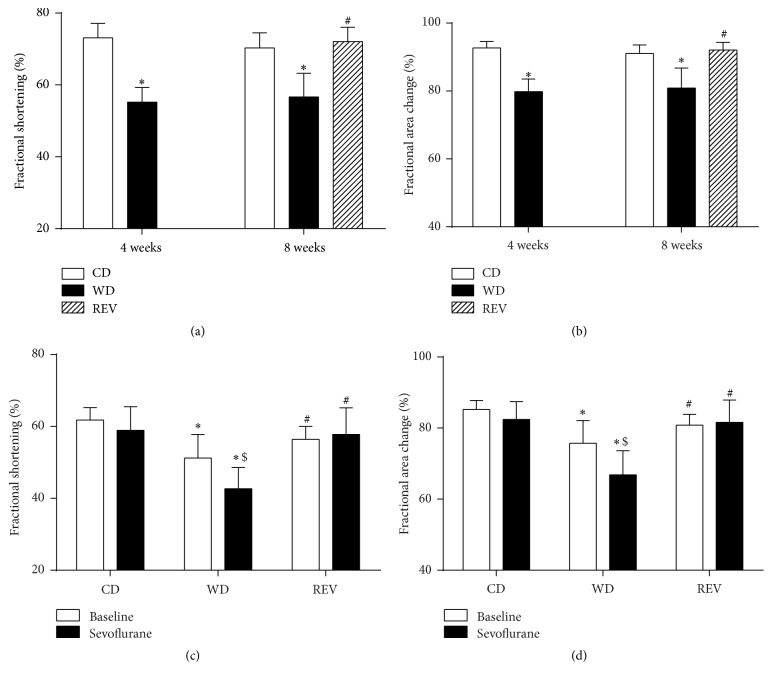
Systolic function after diet feeding with and without sevoflurane exposure. Fractional shortening (a) and fractional area change (b) in rats after 4 and 8 weeks of control diet (CD), western diet (WD), or diet reversion (REV) feeding. Fractional shortening (c) and fractional area change (d) during baseline and sevoflurane exposure in rats after 8 weeks of control diet (CD), western diet (WD), or diet reversion (REV) feeding. Data are mean ± SD, *n* = 6, two-way ANOVA with Bonferroni post hoc analyses, ^*∗*^*p* < 0.05 versus CD, ^#^*p* < 0.05 versus WD, and ^$^*p* < 0.05 versus baseline.

Four weeks of WD-feeding significantly decreased diastolic lumen diameter and increased diastolic wall thickness compared to control rats (Table 2). During end-systole, lumen diameter was significantly increased after WD-feeding, while systolic wall thickness was reduced compared to controls. Systolic functional abnormalities in WD-fed rats were demonstrated by significant reduced fractional shortening and fractional area change after 4 weeks of WD-feeding (Figures 3(a) and 3(b)). Impaired left ventricular relaxation was suggested by significant prolonged E deceleration time and isovolumic relaxation time in WD-fed rats.

After 8 weeks of diet exposure, similar alterations in diastolic and systolic lumen diameter and diastolic wall thickness were found compared to eight weeks of CD-feeding. These alterations were paralleled by impaired systolic function and left ventricular relaxation in WD-fed rats. Diet reversion from WD to CD improved systolic lumen diameter and diastolic wall thickness compared to WD-fed rats (Table 2). Systolic functional abnormalities and deceleration time of the E peak were normalized to control levels after diet reversion (Figures 3(a) and 3(b)).

### 3.3. Sevoflurane Further Impaired Systolic Function during Western Diet Feeding

As shown in Figures 3(c) and 3(d), exposure to sevoflurane did not affect systolic function, as represented by fractional shortening and fractional area change in CD-fed rats. However, sevoflurane further impaired systolic function in WD-fed rats. Diet reversion completely restored the cardiodepressive effects of sevoflurane in WD-fed rats as shown by improved fractional shortening and fractional area change.

### 3.4. Western Diet Feeding Reduces Myocardial Ischemic Injury

**Figure 4:**
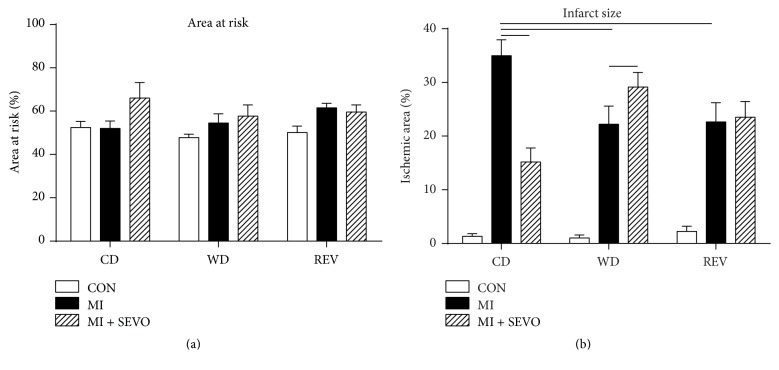
Myocardial ischemia and reperfusion injury. Area at risk (a) and infarct size (b) after sham (CON), myocardial infarction (MI), and myocardial infarction with sevoflurane (MI + SEVO) in rats after 8 weeks of control diet (CD), western diet (WD), or reversion diet (REV) feeding. Data are mean ± SD, *n* = 8–11, one-way or two-way ANOVA with Bonferroni post hoc analyses, ^—^*p* < 0.05.

The area at risk after ischemia and reperfusion did not differ between groups (Figure 4(a)). Unexpectedly, WD-feeding reduced infarct size compared to controls (Figure 4(b)). Interestingly, these protective effects of WD-feeding on ischemic injury persisted after reducing caloric intake (Figure 4(b)).

Hemodynamics at baseline and during myocardial ischemia and reperfusion are given in Table 3. Diet feeding had no effect on blood pressure and heart rate at baseline (one-way ANOVA of CON groups). Myocardial ischemia did not affect blood pressure and heart rate in all diet groups (two-way ANOVA of MI versus CON during ischemia). During the reperfusion phase, diastolic blood pressure and mean arterial pressure were significantly reduced in rats that underwent caloric restriction (REV) (two-way ANOVA of MI versus CON during reperfusion).

### 3.5. Western Diet Feeding Blocked the Cardioprotective Effects of Sevoflurane Anesthesia

**Figure 5:**
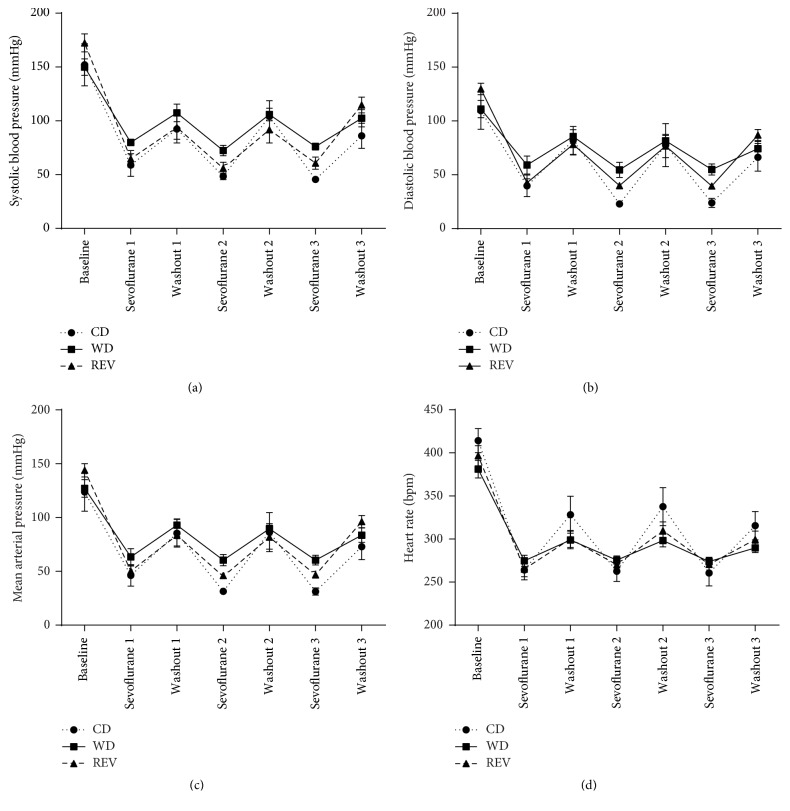
Effect of sevoflurane on hemodynamics. Systolic blood pressure (a), diastolic blood pressure (b), mean arterial pressure (c), and heart rate (d) at baseline and after 3 × 5-minute exposure to sevoflurane with 3 × 5-minute washout periods in between in rats after 8 weeks of control diet (CD), western diet (WD), or reversion diet (REV) feeding. Data are mean ± SD, *n* = 7–10.

Exposure to sevoflurane reduced infarct size in CD-fed rats but offered no additional protection in WD- and REV-fed rats (Figure 4(b)). In particular, infarct size was even bigger in WD-fed rats compared to control rats after sevoflurane exposure. Blood pressure and heart rate decreased during sevoflurane exposure but were partly restored during the washout periods without differences among diet groups (Figure 5). Exposure to sevoflurane had no effect on blood pressure and heart rate during myocardial ischemia and reperfusion in all diet groups (Table 3) (two-way ANOVA of MI versus MI + SEVO during ischemia or reperfusion).

## 4. Discussion 

The present study showed that short-term WD-feeding in rats resulted in a mild T2DM phenotype paralleled by systolic and diastolic dysfunction. Unexpectedly, myocardial infarct size was smaller after WD-feeding. In rats with WD-induced mild T2DM, sevoflurane increased infarct size. Reducing caloric intake restored the WD-induced mild T2DM phenotype and myocardial dysfunction. The protective effects of WD-feeding on infarct size persisted after reducing caloric intake and the cardioprotective effects of sevoflurane anesthesia on ischemic injury were partly restored. These findings suggest that reducing caloric intake in obesity or T2DM might be a possible intervention to reduce perioperative risk of cardiovascular morbidity.

The present study showed that 4 and 8 weeks of WD-feeding resulted in a mild T2DM phenotype and systolic and diastolic dysfunction. Interestingly, reversion of WD after 4 weeks to control diet resulted in reduction of caloric intake and completely normalized the WD-induced mild T2DM phenotype. In small rodents, reduction of caloric intake has been shown to reverse diet-induced obesity [3, 4] and T2DM [19]. However, also contrasting results have been found [20]. Importantly, our study showed that reducing caloric intake also improved diet-induced systolic as well as diastolic dysfunction, which is in agreement with the clinical observations in patient with obesity [5] and T2DM [6], where a low caloric diet decreased myocardial fatty acid uptake [5] and improved diastolic function [6], respectively. Taken together, reducing caloric intake completely restored the mild T2DM phenotype induced by short-term WD-feeding and restored myocardial dysfunction.

Interestingly, the present study demonstrated that infarct size was smaller after WD-feeding. We are not unique with respect to this unexpected finding, as other groups also showed cardioprotective properties of diet feeding during ischemia and reperfusion [21, 22]. On the other hand, also no effect [23, 24] or aggravation [25, 26] of ischemic injury after diet feeding is shown. These conflicting findings may be due to the type and severity of metabolic disease [27], the used ischemia and reperfusion protocol, the duration of diet feeding, and the composition of the diet.

A possible explanation of the reduction in infarct size after WD-feeding is the presence of a high percentage of sucrose [21, 22]. However, this research did not focus on the cardioprotective properties of WD-feeding but on the effect of caloric reduction on ischemic injury. Interestingly, after reducing caloric intake the cardioprotective effect persisted. However, also caloric restriction itself has cardioprotective properties [28].

The volatile anesthetic, sevoflurane, is known for its cardiodepressive effects. The present study showed that sevoflurane further impaired systolic function in WD-fed rats. Interestingly, data are only available on the effects of sevoflurane or other volatile anesthetics in T1DM rats showing smaller [29, 30], greater [31], or unaltered [32] negative inotropic effects. We previously showed that sevoflurane has cardiodepressive effects in WD-induced mild T2DM rats, without a further decrease in myocardial perfusion [9]. Interesting is that these cardiodepressive effects of sevoflurane could be completely restored by reducing caloric intake, suggesting that caloric restriction might be a possible lifestyle intervention to reduce perioperative alterations in myocardial function.

The present study also demonstrated that sevoflurane reduced infarct size in healthy rats but did not have any additive cardioprotective effect in WD-fed rats nor was affected by reducing caloric intake. It has been shown previously that hyperglycemia [13] and obesity/insulin resistance [14] abolished the cardioprotective effect of sevoflurane. However, the generalizability of these models is limited. In diet-induced obesity the cardioprotective effects of sevoflurane were also suppressed [12], whereas in diet-induced early T2DM rats sevoflurane still had cardioprotective properties [33]. Interestingly, our study shows that sevoflurane even increased infarct size in WD-fed rats, suggesting that WD-feeding has negative effects on the cardioprotective properties of sevoflurane. Taken together, the above described results suggest that obesity and/or T2DM affects the cardioprotective effects of sevoflurane but that reducing caloric intake does not affect the protective effect of diet feeding nor the cardioprotective effects of sevoflurane.

These results provide more insight in the positive as well as the negative effects of dietary intake on myocardial function and ischemia and reperfusion injury and the cardioprotective properties of sevoflurane anesthesia. Although difficult to extrapolate to the clinic, these results implicate that the clinician should be aware that lifestyle, dietary intake, and/or obesity might affect perioperative risk. This is supported by the obesity paradox, which showed that patients with underweight or morbid obesity have the highest postoperative morbidity [33]. Future research is necessary to gain more insight into the specific effects of dietary intake and/or BMI on perioperative myocardial function and ischemia and reperfusion injury to create a tailored approach including dietary strategies in order to reduce perioperative risk.

## 5. Conclusions

In conclusion, WD-feeding resulted in a mild T2DM phenotype with systolic and diastolic dysfunction but also had an unexpected cardioprotective effect during myocardial infarction in rats. Sevoflurane anesthesia lost its cardioprotective properties during WD-feeding. Reduction of caloric intake restored myocardial function and the negative effects of sevoflurane, while the cardioprotective effects of WD-feeding persisted. These findings suggest that reducing caloric intake in obesity or T2DM might be a possible intervention to reduce perioperative risk of cardiovascular morbidities.

## Figures and Tables

**Table 1:** Characteristics after 4 and 8 weeks of diet intervention without sevoflurane exposure.

	4 weeks		8 weeks		
	Control diet	Western diet	Control diet	Western diet	Reversion
Caloric intake [kcal/100 gBW]	139 ± 9	145 ± 11	125 ± 6	128 ± 8	115 ± 6^#^
Plasma glucose [mmol/L]	7.0 ± 0.6	7.4 ± 0.8	8.7 ± 0.7	10.5 ± 1.0^*∗*^	8.2 ± 1.2^#^
Plasma insulin [pmol/L]	400 ± 142	691 ± 205^*∗*^	1029 ± 291	1529 ± 381^*∗*^	813 ± 369^#^
Plasma free fatty acids [mmol/L]	0.90 ± 0.18	1.00 ± 0.16	0.28 ± 0.10	0.43 ± 0.09	0.28 ± 0.11
Plasma triglycerides [mmol/L]	0.92 ± 0.21	2.04 ± 0.72^*∗*^	0.71 ± 0.16	3.36 ± 1.28^*∗*^	0.76 ± 0.28^#^
Plasma HDL cholesterol [mg/dL]	71.0 ± 3.0	39.9 ± 9.0^*∗*^	115.7 ± 7.5	62.5 ± 7.0^*∗*^	109.3 ± 12.5^#^
Plasma LDL/VLDL cholesterol [mg/dL]	12.4 ± 2.7	10.2 ± 4.2	16.2 ± 1.7	25.7 ± 5.7^*∗*^	18.0 ± 2.8^#^
Hematocrit [%]	48.3 ± 2.8	47.2 ± 2.9	51.6 ± 2.8	48.1 ± 2.6^*∗*^	49.0 ± 1.3
Bodyweight [g]	368 ± 13	409 ± 6^*∗*^	419 ± 15	476 ± 26^*∗*^	433 ± 30^#^
Heart weight [g]	n.d.	n.d.	1.20 ± 0.06	1.34 ± 0.16^*∗*^	1.29 ± 0.07
Liver weight [g]	n.d.	n.d.	11.0 ± 0.5	14.2 ± 1.1^*∗*^	11.2 ± 2.2^#^
Epididymal fat weight [g]	n.d.	n.d.	6.3 ± 0.3	11.4 ± 2.2^*∗*^	7.4 ± 1.6^#^
Perirenal fat weight [g]	n.d.	n.d.	8.5 ± 0.4	16.3 ± 3.9^*∗*^	9.4 ± 1.9^#^
Tibia length [mm]	n.d.	n.d.	42.0 ± 0.5	41.8 ± 1.1	41.3 ± 1.1

Data are mean ± SD, *n* = 7,4 weeks: Student's *t*-test, ^*∗*^*p* < 0.05 versus control diet; 8 weeks: one-way ANOVA with Bonferroni post hoc test, ^*∗*^*p* < 0.05 versus control diet, ^#^*p* < 0.05 versus western diet, and n.d.: nondetermined.

**Table 2:** Myocardial function after 4 and 8 weeks of diet intervention without sevoflurane exposure.

	4 weeks		8 weeks		
	Control diet	Western diet	Control diet	Western diet	Reversion
Heart rate [bpm]	465 ± 39	486 ± 28	431 ± 26	489 ± 18^*∗*^	474 ± 16
LV mass [mg]	786 ± 95	840 ± 46	846 ± 114	936 ± 72	816 ± 58^#^
*LV dimensions*					
Diastolic lumen diameter [mm]	6.6 ± 0.4	6.1 ± 0.4^*∗*^	6.6 ± 0.5	5.9 ± 0.3^*∗*^	6.1 ± 0.5
Systolic lumen diameter [mm]	1.7 ± 0.2	2.8 ± 0.3^*∗*^	2.1 ± 0.3	2.5 ± 0.3^*ǂ*^	1.7 ± 0.3^#^
Diastolic wall thickness [mm]	1.7 ± 0.1	2.0 ± 0.1^*∗*^	1.7 ± 0.1	2.2 ± 0.2^*∗*^	1.9 ± 0.2^#^
Systolic wall thickness [mm]	3.7 ± 0.2	3.4 ± 0.2^*∗*^	3.7 ± 0.2	3.6 ± 0.1	3.8 ± 0.2
*LV diastolic function*					
E deceleration time [ms]	23.9 ± 7.4	31.0 ± 4.9^*ǂ*^	25.0 ± 2.7	30.2 ± 5.4^*∗*^	23.4 ± 6.3^#^
Isovolumic relaxation time [ms]	13.8 ± 2.3	20.3 ± 4.2^*∗*^	n.d.	n.d.	n.d.

Data are mean ± SD, *n* = 7 and 4 weeks: Student's *t*-test, ^*∗*^*p* < 0.05 versus control diet, ^*ǂ*^*p* = 0.06 versus control diet; 8 weeks: one-way ANOVA with Bonferroni post hoc analyses; ^*∗*^*p* < 0.05 versus control diet, ^*ǂ*^*p* = 0.06 versus control diet, and ^#^*p* < 0.05 versus western diet. LV: left ventricular.

**Table 3:** Hemodynamics during myocardial ischemia and reperfusion after 8 weeks of diet intervention.

	Control diet			Western diet			Reversion		
	CON	MI	MI + SEVO	CON	MI	MI + SEVO	CON	MI	MI + SEVO
*Baseline*									
SBP [mmHg]	137 ± 32	157 ± 31	152 ± 44	134 ± 47	143 ± 22	150 ± 22	152 ± 31	152 ± 40	171 ± 28
DBP [mmHg]	108 ± 36	132 ± 22	110 ± 39	93 ± 39	112 ± 21	111 ± 23	112 ± 25	115 ± 36	128 ± 18
MAP [mmHg]	116 ± 29	138 ± 20	124 ± 40	107 ± 42	122 ± 21	124 ± 22	125 ± 27	128 ± 37	143 ± 21
HR [bpm]	367 ± 64	405 ± 36	414 ± 32	367 ± 67	403 ± 18	381 ± 29	395 ± 49	390 ± 43	397 ± 39
*Ischemia*									
SBP [mmHg]	108 ± 26	104 ± 20	83 ± 28	122 ± 12	107 ± 10	102 ± 11	118 ± 22	100 ± 25	100 ± 25
DBP [mmHg]	74 ± 23	74 ± 13	55 ± 11	65 ± 4	68 ± 14	60 ± 10	69 ± 13	60 ± 12	56 ± 15
MAP [mmHg]	83 ± 14	82 ± 4	64 ± 17	84 ± 6	81 ± 11	74 ± 8	85 ± 14	73 ± 14	70 ± 16
HR [bpm]	276 ± 54	287 ± 22	283 ± 25	294 ± 22	301 ± 39	297 ± 27	271 ± 26	294 ± 39	283 ± 33
*Reperfusion*									
SBP [mmHg]	114 ± 31	91 ± 24	69 ± 24	110 ± 24	91 ± 33	93 ± 18	125 ± 30	92 ± 27	97 ± 13
DBP [mmHg]	82 ± 32	63 ± 18	55 ± 9	72 ± 17	60 ± 30	56 ± 12	82 ± 26	54 ± 17^*∗*^	58 ± 16
MAP [mmHg]	91 ± 28	71 ± 15	59 ± 14	85 ± 18	71 ± 30	68 ± 12	96 ± 26	66 ± 19^*∗*^	71 ± 13
HR [bpm]	286 ± 46	268 ± 16	289 ± 20	292 ± 24	303 ± 60	309 ± 20	302 ± 34	277 ± 29	288 ± 32

Data are mean ± SD, *n* = 7–10, two-way ANOVA with Bonferroni post hoc analyses, and ^*∗*^*p* < 0.05 MI versus CON.

SBP: systolic blood pressure; DBP: diastolic blood pressure; MAP: mean arterial blood pressure; HR: heart rate.
